# The opioid flexion phenotype: postural flexion and nodding in the fentanyl era

**DOI:** 10.3389/fnins.2026.1910436

**Published:** 2026-09-10

**Authors:** Ovie Martin Albert, Hussain Aboud, Alexander Arthur, Ivor Oghenekpavwe Orukpe

**Affiliations:** 1 Department of Family Medicine, Cumming School of Medicine, University of Calgary, Calgary, AB, Canada; 2 Addiction and Mental Health, Centennial Centre for Mental Health and Brain Injury, Recovery Alberta, Ponoka, AB, Canada; 3 Department of Academic Family Medicine, College of Medicine, University of Saskatchewan, Saskatoon, SK, Canada; 4 The Opioid Addiction Treatment Clinic (OAT Clinic), Regina, SK, Canada

**Keywords:** fentanyl, fentanyl nod, harm reduction, medetomidine, naloxone, opioid flexion phenotype, opioid overdose, xylazine

## Abstract

The fentanyl era has produced recurrent community observations of people remaining upright or semi-upright in sustained forward flexion of the head and trunk with intermittent nodding, colloquially referred to as the “fentanyl nod” or “fentanyl fold.” Yet the motor state producing this appearance has never been measured directly. We conducted a structured narrative review spanning axial postural-control and reticulospinal neurophysiology, mu-opioid pharmacology, fentanyl-associated rigidity, alpha-2 adulterants, and observations from supervised consumption settings, and integrated these literatures into a testable framework. We introduce opioid flexion phenotype as a descriptive term for the observed configuration, deliberately holding it apart from any diagnostic or mechanistic claim. We advance disproportionate impairment of axial antigravity control as the primary hypothesis, set against three alternatives the evidence does not yet exclude: generalized sedation with loss of postural support; flexor-predominant rigidity or dystonia; and a mixed or fluctuating motor state. The hypothesis predicts that axial extensor activity will be reduced beyond that expected for the level of arousal at comparable ventilation, without increased flexor co-contraction or passive resistance. The rigidity literature prevents visual flexion from being equated with hypotonia, while alpha-2 agonists such as xylazine and medetomidine are better understood as state-dependent modifiers because they may deepen sedation, alter postural support or attenuate an established rigidity state. Persistent flexion after naloxone is non-specific and does not identify either co-exposure or motor mechanism. Discrimination among the accounts requires concurrent assessment of axial muscle activity, passive tone, arousal, ventilation, kinematics, toxicological exposure and longitudinal response. The immediate clinical and harm reduction implications are mechanism-independent: forward flexion should prompt assessment for toxicity rather than dismissal as sleep or voluntary posture; ventilation, not posture, governs urgency and naloxone titration in suspected opioid overdose; and neither appearance nor naloxone response identifies the underlying motor state. The flexed presentation has been read confidently as sedation, hypotonia and rigidity; none of these interpretations is yet warranted, and the instruments needed to adjudicate among them exist, while the observational protocol does not.

## 1 Introduction

The fentanyl era has altered how community opioid overdose presents. Classical teaching centers on miosis, respiratory depression and unresponsiveness, yet supervised consumption sites and street-level observers increasingly encounter motor and postural presentations that do not fit this template, including sustained forward flexion of the head and trunk. This divergence has emerged as illicitly manufactured fentanyl displaced heroin and alpha-2 adulterants such as xylazine and medetomidine entered the supply, making community overdose more pharmacologically heterogeneous and clinically less predictable (Ciccarone, 2021; Friedman et al., 2022).

Among the atypical presentations reported in fentanyl-era settings is a recurrent postural pattern in which a person remains upright or semi-upright with the head and trunk flexed forward, intermittently regains a more upright position, and then lapses again into flexion. Colloquial terms include the “fentanyl fold” and “fentanyl nod,” while related descriptions such as the “heroin hunch” predate the current fentanyl era. Although this configuration has been documented in community and clinical observations, the motor state that produces it remains unresolved. It is commonly read as severe sedation, has been recorded within descriptions of fentanyl-associated rigidity, and has more recently been framed as a possible drug-induced movement disorder (Ganos et al., 2025; Kinshella et al., 2018). These interpretations are not equivalent: each implies a different relationship among arousal, muscle tone, motor control and clinical response. These differing interpretations have accumulated as the drug supply itself has changed, making the contemporary pharmacological context essential to understanding why the presentation is now being encountered.

A 2018 observational report of more than 1,500 overdose responses at a Vancouver supervised injection site documented atypical presentations in roughly one-third of cases (Kinshella et al., 2018). Among the muscle rigidity cases, observers recorded flexed posturing, including clients found with a flexed posture and clenched hands while non-responsive (Kinshella et al., 2018). That work established that fentanyl-era overdose departs substantially from classical opioid teaching, but it recorded these observations within a rigidity category and did not resolve whether the posture reflects reduced or increased muscle tone. A rapid ethnographic study in the same city independently described body and chest rigidity as distinguishing fentanyl-related overdose (Mayer et al., 2018). Separately, medical-examiner and survivor-interview data from Massachusetts described atypical features of fentanyl overdose including sudden rigidity, rapid loss of consciousness and early cyanosis (Somerville et al., 2017). More recently, a movement-disorders group has described a related forward-flexed posture as a candidate drug-induced movement disorder, proposing links to camptocormia and to basal-ganglia and dopaminergic mechanisms, and noting that in some individuals the posture persists after drug use has stopped (Ganos et al., 2025).

Several features of the contemporary supply bear on this. Fentanyl differs from heroin in ways that matter clinically: high mu-opioid receptor potency, rapid central nervous system penetration owing to lipophilicity, and a short onset-to-peak interval produce abrupt state shifts and narrow the window between use and clinical effect (Ciccarone, 2021; Comer and Cahill, 2019; Suzuki and El-Haddad, 2017). It is now the dominant opioid detected in United States overdose deaths and is involved in most accidental apparent opioid-toxicity deaths in Canada (Public Health Agency of Canada, 2025; Tanz et al., 2024), so these properties are encountered at population scale rather than in isolated exposures.

The supply is also unstable and increasingly polysubstance. Seized-drug analyses in British Columbia showed strong correlations between fentanyl seizures and overdose deaths, and increasing co-presence of fentanyl with other opioids rather than circulation as a single predictable exposure (Baldwin et al., 2018). These findings underscore substantial heterogeneity in the opioid composition of seized samples. Xylazine, a veterinary alpha-2 adrenergic agonist reported as an emerging drug of abuse in Puerto Rico (Reyes et al., 2012), spread across North America from 2019 and is now documented in a substantial proportion of fentanyl-involved deaths in many jurisdictions (Friedman et al., 2022; Gupta et al., 2023). Medetomidine, an alpha-2 agonist with approximately 100-fold greater alpha-2 receptor affinity than xylazine (Schwartz and Clark, 1998), has since emerged in the illicit supply (Schwarz et al., 2024; Sisco and Appley, 2023), reaching 72% of tested Philadelphia illicit opioid samples by late 2024 (Huo et al., 2025). The pharmacological substrate of community overdose is therefore neither the heroin-era substrate nor a fentanyl-only substrate, and the presentations that follow should not be expected to track either template.

This Hypothesis and Theory article addresses what the flexed posture represents. We separate the observation from the inference and from the proposed mechanism, set out the candidate accounts that remain consistent with the available evidence, and specify the measurements that would distinguish them. We use the term flexion phenotype descriptively, as shorthand for an observed postural pattern, and not as a defined diagnostic or nosological entity. Our primary mechanistic hypothesis is that axial antigravity control is impaired disproportionately to the level of arousal at comparable ventilation; we examine it alongside generalized sedation, flexor-predominant rigidity or dystonia, and mixed motor states, and we treat alpha-2 adulterants as context-dependent modifiers rather than as defining causes.

The gap this article addresses is not the absence of observation but the absence of measurement. Fentanyl-era surveillance has documented that atypical motor presentations are common, but to our knowledge no published study has measured axial muscle activity or tone against arousal at comparable ventilation during illicit fentanyl exposure, and without that measurement the candidate accounts cannot be separated. The presentation therefore sits between literatures that have not been connected: the neurophysiology of axial postural control, the brainstem pharmacology of mu-opioid and alpha-2 agonism, the anesthetic literature on fentanyl-associated rigidity, and the observational record from supervised consumption settings. We move from established posture and arousal physiology (§3.2 and §3.3), through the modifier pharmacology (§3.4) and the competing accounts and the measurements that would discriminate among them (§3.5), to the clinical and research implications (§4 and §5). The clinical recommendations that follow hold across these competing accounts, since they rest on ventilation and observable clinical features rather than on any single mechanistic interpretation.

## 2 Methods

### 2.1 Review approach and rationale

We undertook a structured narrative review integrating evidence from motor and postural-control neurophysiology, mu-opioid receptor pharmacology, anesthesia and toxicology case literature, animal models of fentanyl-analogue neurorespiratory toxicity, observational studies from supervised consumption settings, and forensic and surveillance reports on alpha-2 adulterants. These literatures differ in design, scale and evidentiary purpose, but each addresses a distinct part of the same clinical and mechanistic problem. We used a narrative, hypothesis-generating synthesis (Chaney, 2021; Sukhera, 2022), connecting established physiology, experimental pharmacology, clinical observation and drug-supply surveillance through explicit mechanistic bridging. The review process was informed by published guidance on narrative review methodology (Agarwal et al., 2023; Gasparyan et al., 2011; Green et al., 2006), and reporting quality was self-assessed using the Scale for the Assessment of Narrative Review Articles (SANRA; Supplementary Material 3) (Baethge et al., 2019).

### 2.2 Search strategy

We searched PubMed, Embase, and PsycINFO for English-language literature published from database inception to July 2026, with selected foundational publications from earlier decades included where they remained the canonical references for posture neurophysiology and reticulospinal organization. Search strings combined controlled vocabulary and free-text terms across five thematic clusters: postural control and reticulospinal neurophysiology; mu-opioid receptor pharmacology, fentanyl, and brainstem respiratory and motor control, including opioid action at motoneurons; xylazine, medetomidine, and other alpha-2 adrenergic agonists in the illicit drug supply; observational studies of fentanyl-era overdose presentations including atypical motor phenomena and supervised consumption site surveillance data; and drug-induced movement disorders and axial postural abnormality. Reference lists of major reviews, public health surveillance reports, and clinical case literature on fentanyl-associated rigidity were hand-searched for additional sources, an approach shown to identify substantial additional evidence in reviews of complex evidence (Greenhalgh and Peacock, 2005). Forward citation-tracking was conducted from the foundational neurophysiological and primary rigidity literature. The full database search strategy is provided in Supplementary Material 1.

### 2.3 Source selection and prioritization

Sources were prioritized according to an explicit evidence hierarchy. Human observational and pharmacological data were given primacy for clinical claims about overdose phenotype and naloxone responsiveness. Established neurophysiological literature on reticulospinal organization and postural control was given primacy for mechanistic claims about axial tone regulation. Anesthesia case literature on fentanyl-induced rigidity was used to characterize the contrasting wooden chest phenotype and to support the competing accounts of the flexed presentation. CDC and public health surveillance reports were used for current characterization of the alpha-2 adulterant supply. Animal-model studies of fentanyl analog neurorespiratory toxicity were used to support mechanistic plausibility of dose-effect relationships in concurrent respiratory and postural depression but were not used to support direct clinical claims about human overdose presentations. Where reports diverged, we describe the divergence rather than adjudicate between them. The full source selection criteria and evidence hierarchy are provided in Supplementary Material 2.

### 2.4 Synthesis

Evidence was synthesized across two thematic domains. The mechanistic-basis domain (§3) integrates posture neurophysiology, opioid pharmacology, alpha-2 adulterant pharmacology, and the competing accounts of the flexed presentation; the clinical-implications domain (§4) addresses recognition, naloxone strategy, and harm reduction practice. The evidence informing each thematic domain, and the points at which it conflicts or remains unresolved, are summarized in Table 1. Where direct human data linking illicit fentanyl exposure to sustained postural flexion were limited, we explicitly applied mechanistic bridging, distinguishing convergent inference from established empirical findings. This review did not involve human participants and did not require ethical approval.

**TABLE 1:** Evidence informing the framework, by domain: what is established and where the evidence conflicts.

Domain	What the evidence establishes	Where the evidence conflicts or is unresolved	Principal source types
Postural control and reticulospinal organization	Upright posture is actively maintained by tonic descending drive to axial musculature; medial reticulospinal projections are excitatory with an extensor bias, lateral medullary projections inhibitory (Deliagina et al., 2006; Peterson et al., 1979; Takakusaki, 2017)	Atonia-related and hypertonus-related reticulospinal populations occupy distinct territories, so suppression need not shift tone in a single direction (Takakusaki et al., 2016). None of this literature involves opioid exposure	Foundational neurophysiology; primary animal electrophysiology
Spinal noradrenergic architecture	Spinally projecting locus coeruleus neurons are concentrated ventrally and caudally, descending in the ipsilateral ventral funiculus alongside the A5 and A7 groups (Loughlin et al., 1986a,1986b; Westlund et al., 1984)	Directly disputed and method-dependent: substantial ventral-laminae terminations reported (Clark and Proudfit, 1991) against selective sparing of A5 and A7 after coerulospinal lesioning (Lyons et al., 1989). The direction of the fentanyl effect on locus coeruleus output is itself contested (Torralva and Janowsky, 2019)	Anterograde tracing; immunocytochemistry; selective neurotoxin lesioning
Brainstem mu-opioid pharmacology	Fentanyl depresses respiratory rhythm through preBötzinger and Kölliker-Fuse mu-opioid receptor populations (Bachmutsky et al., 2020; Pattinson, 2008; Saunders and Levitt, 2020)	Arousal and respiratory rate depression are related but not identical, with respiratory depression correlating with cortical arousal reduction in rats given fentanyl and in children given morphine (Montandon and Horner, 2019; Montandon et al., 2016), and rigidity mediated by a different receptor population from respiratory depression (Yang et al., 1992). Rigidity, respiration and sedation dissociate pharmacologically (Lewis and Haouzi, 2025)	Primary rodent electrophysiology; targeted receptor deletion; human electrocortical studies
Opioid action at spinal motor circuits	Mu- and delta-opioid receptor co-expression predominates in ventral horn motor circuits (Wang et al., 2018); mu-opioid receptor agonism at a brainstem motor pool depresses output to its target muscle (Hajiha et al., 2009)	Functional direction is not settled: intrathecal morphine increases hindlimb muscle tone after mild spinal cord injury (Kawakami et al., 2022) while a mu-opioid receptor agonist reduces excitatory drive to lamina IX motoneurons (Wu et al., 2017). No measurement of reticulospinal or axial postural output under opioid exposure has been reported	Receptor mapping; spinal electrophysiology; intrathecal pharmacology
Fentanyl-associated muscle rigidity	Rigidity extends to the limbs and trunk, including trunk flexors, and is common at high dose and rapid administration (Freund et al., 1973; Grell et al., 1970; Haouzi and Tubbs, 2022; Lui et al., 1989; Streisand et al., 1993); repeated high-dose exposure shows no significant attenuation across ten exposures (Obert et al., 2025)	Mechanism unresolved: coerulospinal noradrenergic and spinal alpha-1 pathways (Fu et al., 1997; Lui et al., 1990, 1993, 1995) alongside a mu-opioid-receptor-independent potassium channel mechanism (Wei et al., 2026). Naloxone response is variable (Çoruh et al., 2013; Miner et al., 2021)	Anaesthesia case literature; human volunteer studies; primary rodent electromyography and channel physiology
Alpha-2 adulterants as modifiers	Xylazine is widespread in the North American illicit fentanyl supply, while medetomidine is emerging and has become highly prevalent in some local markets; the sedative effects of both are not reversed by naloxone (Friedman et al., 2022; Gupta et al., 2023; Huo et al., 2025; Kariisa et al., 2023)	The direction of the postural effect is not established. Alpha-2 agonism reduces opioid-induced rigidity in several models (Haouzi and Tubbs, 2022; Weinger et al., 1989, 1995), and xylazine at a human-relevant dose reduced fentanyl-induced respiratory muscle rigidity (Lewis et al., 2026b). In one prospective cohort, alpha-2 detection was not associated with prolonged sedation (Love et al., 2026a); in a separate analysis, bradycardia was associated with xylazine detection (Love et al., 2026b); and a pilot study found similar pre-hospital naloxone dosing with and without detected xylazine (Merchant et al., 2026)	Public health surveillance; forensic detection; primary rodent pharmacology; prospective clinical cohorts
Observation of the presentation	Atypical motor presentations are common in fentanyl-era overdose; muscle rigidity was the single most frequent, in 240 of 1,581 responses (Kinshella et al., 2018); the proportion of events involving rigidity rose from 10.4% to 18.9% as fentanyl entered the supply (Notta et al., 2019); and a separate ethnographic study described body and chest rigidity as distinguishing fentanyl-related overdose (Mayer et al., 2018)	Flexed posturing was recorded within a rigidity category, so the observational record does not establish whether tone is reduced or increased. The same posture has separately been framed as a drug-induced movement disorder (Ganos et al., 2025)	Supervised consumption site observation; rapid ethnography; medical examiner data; movement-disorder clinical description

## 3 Mechanistic basis of the flexed presentation

### 3.1 Working operational description

**FIGURE 1:**
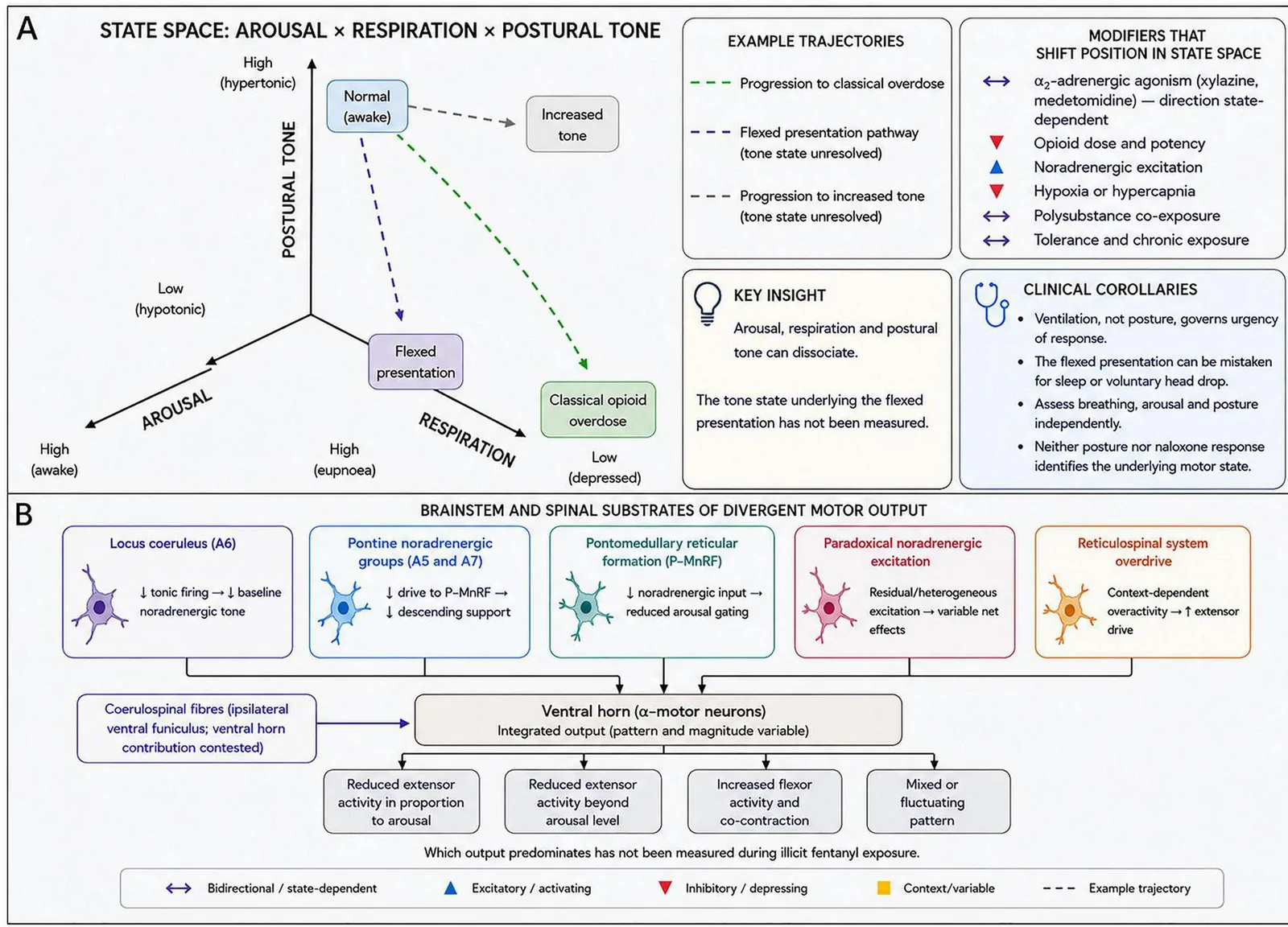
Neurophysiological state space and brainstem substrates of the flexed presentation. Panel **A** locates the flexed presentation within a three-axis state space defined by arousal, respiration and postural tone, showing that these can dissociate; modifiers that shift position within the space are listed alongside. Panel **B** shows the brainstem and spinal substrates that converge on ventral horn motor output, and the four patterns of axial motor output that could result. Anatomical relationships are schematic and not to scale. Which output predominates has not been measured during illicit fentanyl exposure.

The observed posture, the inferred muscle tone state and the proposed mechanism must be distinguished. The observational claim is that people using illicitly manufactured high-potency opioids are seen in sustained forward flexion of the head and trunk, with intermittent partial recovery of the upright position; observers at supervised consumption sites have documented flexed posturing among atypical motor presentations (Kinshella et al., 2018; Notta et al., 2019), and the posture has more recently been described as a candidate drug-induced movement disorder (Ganos et al., 2025). The inferential claim is that the posture reflects reduced tone in the axial antigravity musculature. The mechanistic claim is that any such reduction arises from suppression of tonic reticulospinal and monoaminergic drive to axial motoneurons (Takakusaki, 2017; Takakusaki et al., 2016). Only the postural configuration has been observed directly. Axial tone has not been measured in this population, and the proposed mechanism is the primary hypothesis examined here against competing explanations. The description below is therefore confined to the observational level and treats muscle tone as a variable to be assessed rather than as a defining feature (Figure 1).

For research purposes we propose the following working description. The presentation is characterized by: (1) recent suspected exposure to illicitly manufactured fentanyl or another high-potency opioid; (2) sustained forward flexion of the cervical spine, the trunk, or both, while seated, standing or otherwise upright or semi-upright, with incomplete loss of postural set such that the person does not collapse fully to the ground; (3) intermittent partial restoration of the upright position; (4) fluctuation of the postural state over seconds to minutes within a single episode; and (5) temporal association with the acute exposure episode, without evidence that the same postural configuration was persistently present at baseline.

Three features are excluded from the working criteria: axial hypotonia, the absence of rigid or dystonic posturing, and persistence after opioid antagonism. Neither the tone state nor the distribution of muscle activity has been measured in this population. Fentanyl produces skeletal muscle rigidity extending beyond the chest wall to the limbs and trunk (Haouzi and Tubbs, 2022; Torralva and Janowsky, 2019), including contraction of the abdominal musculature, a trunk flexor, in animal preparations (Lui et al., 1989), with analogous abdominal-wall rigidity described in humans given high-dose morphine (Freund et al., 1973). A forward-flexed posture is therefore compatible with reduced antigravity drive and with flexor-predominant contraction alike, and the community observations drawn on here were originally recorded within a rigidity framing (Kinshella et al., 2018). Persistence after opioid antagonism is likewise non-specific: it does not establish complete opioid reversal, distinguish flexion from sedation or identify alpha-2 co-exposure, and including it as a criterion would make any later appeal to it circular.

The working description is confined to the acute episode. Postural abnormality persisting between exposures, or after cessation of use, has been described in people with prolonged high-potency opioid and stimulant exposure and may involve mechanisms, including basal ganglia disturbance and secondary musculoskeletal change, that may differ from those operating during the acute state (Ganos et al., 2025).

Conditions considered alongside this presentation fall into two groups: clinical mimics that produce a similar posture through unrelated processes and require exclusion, and candidate explanations for the acute configuration itself. The mimics, which include chronic parkinsonian axial flexion, for which consensus nosology and cut-off values exist (Tinazzi et al., 2022), are set out in Table 2. The candidate explanations are generalized sedation with loss of postural support; disproportionate impairment of axial antigravity control; flexor-predominant rigidity or dystonia (Haouzi and Tubbs, 2022; Torralva and Janowsky, 2019); and mixed or fluctuating motor states. Acute basal ganglia or dopaminergic disturbance remains an exploratory possibility, supported by lesion and preclinical evidence but not linked directly to the acute postural presentation (Ganos et al., 2025; Nieves et al., 2001; Trinko et al., 2024). Alpha-2 agonists are treated as context-dependent modifiers capable of influencing more than one of these states rather than as a separate motor phenotype. These are examined in §3.5 rather than excluded by definition.

**TABLE 2:** Considerations for the flexed presentation: conditions to exclude, and candidate explanations to assess.

Group A. Conditions producing a similar posture through unrelated processes (to be excluded)		
**Condition**	**Distinguishing features**	**Bedside or supervised consumption site cue**
Non-opioid sedative intoxication without opioid co-exposure	Sedation and reduced postural support possible, but no opioid exposure history and no response to naloxone	Absence of recent opioid use; no clinical change after naloxone; slurred speech or ataxia where present
Catatonia	Sustained postural holding rather than dynamic flexion-recovery cycling; waxy flexibility; mutism; psychiatric history typical	Posture held against gravity in unusual configurations, not specifically forward-flexed; gegenhalten; absence of opioid exposure
Chronic axial postural abnormality of parkinsonism	Chronic, progressive flexion present at baseline; parkinsonian features; consensus nosology and cut-off values established (Tinazzi et al., 2022)	Flexion documented before recent drug use; resting tremor, bradykinesia; established diagnosis
Cervical myopathy or motor neuron disease (dropped head syndrome)	Chronic neck extensor weakness; flexion present at baseline; not associated with recent exposure or altered consciousness	Persistent head drop without acute intoxication; chronic course documented in clinical history
Stimulant dyskinesia or stereotypy	Hyperkinetic rather than sustained flexed posture; repetitive purposeless movements; preserved or heightened arousal	Picking, repetitive head movements or other stereotypy; tachycardia, mydriasis, agitation rather than sedation
Seizure or postictal state	Tonic-clonic or focal motor activity during the event; postictal confusion with sustained reduced consciousness	Witnessed convulsive activity, tongue biting, incontinence; prolonged sedation unresponsive to naloxone
Syncope	Transient loss of consciousness with rapid recovery; collapse to the ground rather than sustained semi-upright flexion	Brief duration; full recovery within seconds to minutes
Cervical trauma	Acute traumatic mechanism; localized neck pain; possible neurological deficit	Witnessed fall or impact; visible injury; pain on cervical movement; lateralized neurological signs
Group B. Candidate explanations for the flexed presentation (not exclusions)		
**Candidate explanation**	**Basis**	**What would refute it**
Generalized sedation with loss of postural support	Supervised monitoring data describe graded sedation and vital-sign patterns (Wishik et al., 2021); on this account postural change tracks arousal and no separate mechanism is required	Refuted if axial extensor activity is reduced disproportionately to arousal at comparable ventilation
Disproportionate impairment of axial antigravity control	Reduction of excitatory antigravity output beyond what the level of arousal would predict (§3.2, §3.3)	Refuted if extensor activity tracks arousal, or if flexor co-contraction or passive resistance is increased
Fentanyl-associated flexor-predominant rigidity or dystonia	Rigidity documented in limbs and trunk rather than chest wall alone, including trunk flexors (Freund et al., 1973; Haouzi and Tubbs, 2022; Lui et al., 1989; Torralva and Janowsky, 2019)	Refuted if flexor electromyographic activity, co-contraction and passive resistance are not increased
Mixed or fluctuating motor state	More than one pattern within a single episode; the community record describes flexion and rigidity in the same individuals (Kinshella et al., 2018)	Refuted if serial assessment shows a single stable pattern throughout
Acute basal ganglia or dopaminergic disturbance (exploratory)	Proposed by analogy with camptocormia and pallidal lesion syndromes (Ganos et al., 2025; Nieves et al., 2001); not established for the acute presentation	Would predict features of parkinsonian axial syndromes and looser coupling to the acute exposure episode

Group A is an author-generated clinical differential intended to structure assessment rather than a validated diagnostic algorithm; the bedside cues are illustrative and do not replace standard clinical evaluation. The entries in the final column of Group B are prospective predictions generated by the framework rather than validated diagnostic criteria. Wooden chest syndrome is not listed as a differential. It is the severe ventilatory expression of fentanyl-associated rigidity (§3.5), and rigidity in a flexor-predominant distribution is a candidate explanation for the flexed presentation rather than an alternative to it. Group B cannot be separated by inspection at the bedside; the measurements required are set out in §3.5 and §5.

Each criterion requires operational specification for prospective use, and we propose the following as a starting point.

*Postural configuration* Sustained forward flexion of the head, the trunk or both, while seated or standing, with incomplete loss of postural set. Cervical flexion may dominate, truncal flexion may dominate, or both may co-occur. Lateral bending should be recorded separately, since it has been described in the chronic presentation (Ganos et al., 2025).

*Muscle tone* Axial and appendicular tone should be assessed and recorded rather than inferred from posture: as reduced, increased, mixed, or indeterminate, with the basis for the judgment noted. In trained clinical or research settings, and only after trauma and other contraindications have been excluded, resistance to passive movement of the neck and trunk, the presence of co-contraction, and the distribution of any increased tone across flexor and extensor groups are each informative, and postural tone in axial muscles is distinct from the phasic tone routinely assessed in the limbs (Ganguly et al., 2021). Where formal assessment is not feasible, indeterminate is the correct entry.

*Motor behavior* Intermittent head drop or nodding with reduced or delayed postural correction. Gross body position may be relatively preserved despite marked flexion. Additional motor features, including tremor, stereotypy or fixed abnormal posturing of the limbs, should be recorded rather than treated as excluding the presentation, since the motor phenomenology of fentanyl-era presentations is variable (Ganos et al., 2025; Kinshella et al., 2018; Notta et al., 2019).

*Level of responsiveness* Fluctuating between drowsiness and transient unresponsiveness, with partial arousability to verbal or tactile stimuli. Responsiveness should be recorded separately from postural state and tone, since the three can dissociate: in human volunteers given high-dose fentanyl, rigidity and unresponsiveness occurred together (Streisand et al., 1993).

*Respiratory context* Respiratory rate, depth and regularity should be assessed concurrently. The presentation may occur with slow, shallow or irregular breathing, or with relatively preserved ventilation. Postural state does not specify respiratory state, and respiratory assessment governs the urgency of response (Montandon and Slutsky, 2019; Pattinson, 2008).

*Temporal characteristics* Episodic and fluctuating over minutes, and may progress toward deeper unresponsiveness or improve transiently with stimulation. The duration of any single episode, the pattern of recurrence, and the postural state between episodes are each informative.

*Response to intervention* Change in posture, responsiveness and ventilation after stimulation and opioid antagonism should be recorded separately, because they may not move together. Improvement in ventilation without corresponding recovery of posture or arousal is non-specific and may reflect residual or recurrent opioid toxicity, a non-opioid co-intoxicant, or hypoxic or hypercapnic effects; without toxicological confirmation it establishes none of these and should not be attributed to a particular co-exposure (Love et al., 2026b). Its mechanistic interpretation is addressed in §3.5 and its clinical implications in §4.2.

### 3.2 Upright posture as an active neurophysiological achievement

Standing upright with the head balanced over the trunk is an actively maintained state, not a passive equilibrium. It depends on continuous tonic drive from brainstem reticulospinal pathways to axial and proximal limb musculature, modulated by ongoing integration of vestibular, visual and proprioceptive input (Deliagina et al., 2006; Takakusaki, 2017). The pontine and medullary reticular formation generate background postural muscle tone, the cerebellum provides moment-to-moment error correction, and cortical and basal-ganglia circuits organize anticipatory postural adjustments in advance of voluntary movement (Takakusaki, 2017; Takakusaki et al., 2004). Even quiet standing recruits a distributed cortical-subcortical network (Horak, 2006; Wittenberg et al., 2017). Postural tone is sustained by multiple parallel descending drives – reticulospinal, vestibulospinal and monoaminergic – whose collective output is calibrated against postural demand, gravity and arousal (Cacciatore et al., 2024; Gurfinkel et al., 2006).

The reticulospinal system is not anatomically uniform (Baker, 2011). The medial reticulospinal tract, arising from nucleus reticularis pontis caudalis and the dorsorostral part of nucleus reticularis gigantocellularis, descends ipsilaterally and exerts monosynaptic excitatory drive on axial motoneurons, with an extensor bias for the antigravity muscles of the neck and trunk (Peterson et al., 1979; Takakusaki, 2017). The lateral medullary reticulospinal projection arises from more caudal nuclei and exerts a complementary inhibitory influence on the same extensor pools (Peterson et al., 1979), an inhibitory action demonstrated at the level of single medullary reticulospinal neurons acting through spinal interneurons (Takakusaki et al., 1989). Activation of anatomically differentiated medullary regions favors atonia or hypertonus (Takakusaki et al., 2016), so the system can shift axial tone in either direction. Superimposed on this is distributed pontine noradrenergic modulation involving the locus coeruleus/subcoeruleus and the A5 and A7 cell groups (Clark and Proudfit, 1991; Lyons et al., 1989; Samuels and Szabadi, 2008), which links arousal and sensory-motor gain to spinal motor output (Burgess and Peever, 2013; Witts et al., 2023). Pharmacological suppression therefore need not shift tone in a single direction; its net effect depends on which populations are engaged.

Two consequences follow. Preferential reduction of excitatory antigravity output, rather than non-specific suppression of the reticulospinal system as a whole, could reduce axial extensor support and favor forward flexion (Gurfinkel et al., 2006; Takakusaki et al., 2004). If that reduction were incomplete or fluctuating, it might produce partial loss and recovery of upright posture rather than complete collapse. This is one candidate account of nodding; the sources cited characterize postural physiology in the absence of opioid exposure and do not themselves examine opioid-associated nodding. Distinguishing it from generalized sedation, flexor-predominant rigidity or dystonia, and mixed motor states is taken up in §3.5.

### 3.3 Fentanyl pharmacology at the intersection of arousal, posture, and respiration

Fentanyl is a high-affinity, high-efficacy mu-opioid receptor (MOR) agonist that penetrates the central nervous system rapidly because of its lipophilicity and produces potent respiratory depression, although the intracellular signaling pathways linking exposure to respiratory toxicity remain debated (Bateman et al., 2023). It has a faster onset than morphine and, after a single bolus, a shorter apparent clinical duration owing to rapid redistribution (Comer and Cahill, 2019; Suzuki and El-Haddad, 2017). MOR are widely distributed and mediate effects extending well beyond analgesia (Le Merrer et al., 2009). Relevant brainstem sites include the preBötzinger complex and Kölliker-Fuse/parabrachial complex, which generate and modulate respiratory rhythm (Montandon and Slutsky, 2019; Pattinson, 2008), and the locus coeruleus and adjacent reticular nuclei, which contribute to arousal (Samuels and Szabadi, 2008). Primary work localizes the respiratory effects directly: MOR in the Kölliker-Fuse/parabrachial complex contribute to fentanyl-induced apnea and respiratory rate depression (Saunders and Levitt, 2020), and opioid depression of breathing has been traced to two small brainstem sites encompassing the preBötzinger complex (Bachmutsky et al., 2020).

The conventional account of opioid overdose emphasizes respiratory depression mediated through these networks (Bateman et al., 2023; Pattinson, 2008). Experimental evidence nevertheless indicates that opioid effects on arousal and respiratory rhythm are related but distinguishable: in rats, fentanyl produces an electrocortical state unlike sleep or wakefulness, and the magnitude of respiratory rate depression correlates with the degree of cortical arousal reduction (Montandon and Horner, 2019); a comparable association between sedation-related electrocortical change and respiratory rate depression was observed in children given morphine (Montandon et al., 2016). The dissociation extends to motor output: in rats given high-dose fentanyl, nalfurafine reversed muscle rigidity and restored ventilation to pre-fentanyl levels while the animals remained fully sedated (Lewis and Haouzi, 2025). Any account of opioid-associated postural change must therefore specify which outputs are expected to co-vary and which may dissociate.

**FIGURE 2:**
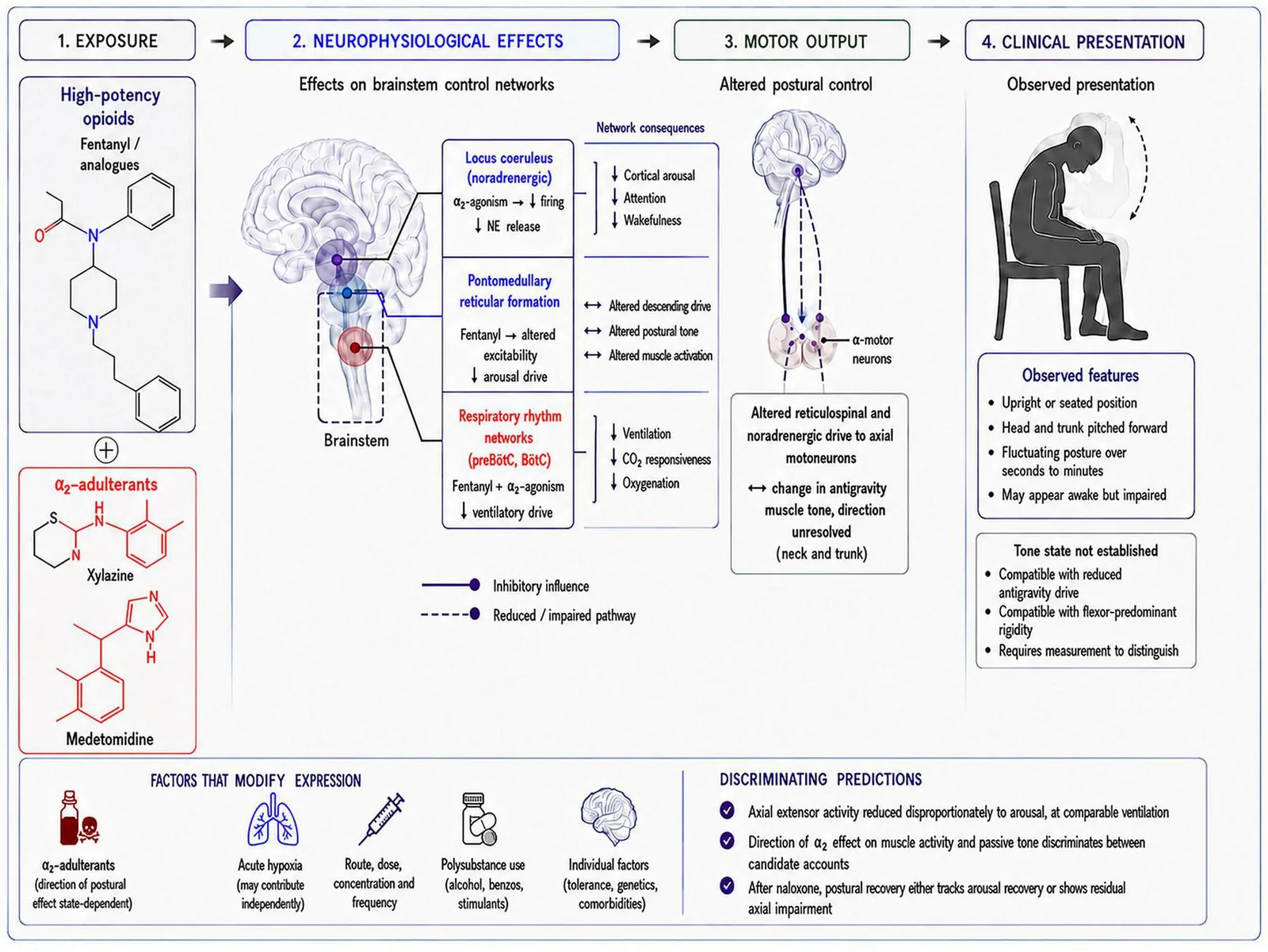
Neurophysiological framework for the flexed presentation. High-potency opioids, alone or with alpha-2 adulterants, act on interconnected brainstem networks regulating arousal, respiration and postural tone. The resulting changes in descending drive could alter axial antigravity control or flexor-extensor balance and thereby contribute to sustained forward flexion with intermittent nodding. The direction and distribution of the tone change have not been measured during illicit fentanyl exposure. The panel summarizes factors that modify expression and the three discriminating predictions that follow.

We propose that MOR activation within neighboring and interconnected pontomedullary networks may, under some exposure conditions, depress axial postural output alongside respiratory control (Figure 2). For this to constitute more than the postural expression of generalized sedation, axial extensor output would need to be reduced disproportionately to the level of arousal at comparable ventilation, which has not been demonstrated and is the principal empirical test of the model. The proposal is supported indirectly by the proximity and interconnection of the relevant pontomedullary networks and by proof of principle that MOR activation can depress output from a brainstem motor pool: direct application of fentanyl to the hypoglossal motor nucleus suppresses genioglossus activity in a naloxone-reversible manner (Hajiha et al., 2009), though that nucleus supplies upper-airway rather than axial postural musculature, an effect implicated in opioid-associated upper airway compromise (Freire et al., 2022). Opioid receptors are also present in spinal motor circuitry, with mu- and delta-opioid receptor co-expression confined to small populations of dorsal horn neurons but predominating in ventral horn motor circuits (Wang et al., 2018). The functional evidence, however, points in both directions: in rats with mild thoracic spinal cord injury, intrathecal morphine increases hindlimb muscle tone through spinal mu- and delta-opioid receptors (Kawakami et al., 2022), whereas a MOR agonist reduces excitatory synaptic drive to lamina IX motoneurons (Wu et al., 2017). Neither reticulospinal drive nor axial postural motor output has been measured during opioid exposure; studies of opioid-induced respiratory depression and brain hypoxia measured respiratory or oxygenation endpoints rather than postural motor output (Kiyatkin, 2019; Montandon and Slutsky, 2019), and the available spinal evidence does not favor a uniformly depressant account.

### 3.4 Alpha-2 adulterants as state-dependent phenotype modifiers

The fentanyl-era supply is increasingly polysubstance, with xylazine, a veterinary alpha-2 adrenergic agonist, now a major adulterant across North America (Friedman et al., 2022; Gupta et al., 2023; Kariisa et al., 2023). Xylazine acts at central alpha-2 adrenoceptors, reducing noradrenergic output, sympathetic tone and central arousal, and its sedative effects are not reversed by naloxone (Gupta et al., 2023); its receptor pharmacology is not exclusively adrenergic, since in rodents it is also a full agonist at kappa opioid receptors (Bedard et al., 2024). These properties make xylazine a plausible modifier of motor expression but do not predetermine the direction of its effect on posture, and an effect on the duration of clinical sedation is not established: in a multicenter prospective cohort of emergency department patients with opioid overdose, alpha-2 adulterant detection was not associated with prolonged sedation (Love et al., 2026a).

A simple additive model would predict that alpha-2 co-exposure deepens and prolongs flexion by further reducing noradrenergic support to the same targets. Experimental evidence instead shows that the motor effect is not uniform. In rat models, dexmedetomidine prevented opioid-induced muscle rigidity through central alpha-2 adrenoceptors (Weinger et al., 1989), antagonized it dose-dependently (Weinger et al., 1995), and in rats made rigid by high-dose fentanyl restored respiratory system compliance and reversed the associated hypermetabolism while the animals remained sedated (Haouzi and Tubbs, 2022). The same direction of effect has been shown for xylazine itself: at 0.1 mg/kg, a dose chosen to match reported human exposures, xylazine reduced fentanyl-induced rigidity of respiratory muscles and oxygen consumption in rats, while higher doses increased mortality dose-dependently (Lewis et al., 2026b). Conversely, the alpha-2 antagonist atipamezole augmented alfentanil-induced rigidity and increased baseline tonic electromyographic activity (Weinger and Bednarczyk, 1994), though an earlier report of alpha-2-mediated reversal is confounded by concurrent alpha-1 antagonism (Jerussi et al., 1987; Torralva and Janowsky, 2019).

Together these findings support a state-dependent rather than uniform effect. Rat models implicate coerulospinal noradrenergic signaling and spinal alpha-1 adrenoceptors in fentanyl-induced rigidity (Lui et al., 1990, 1995; Torralva and Janowsky, 2019), and rigidity and respiratory depression are mediated by different receptor populations (Yang et al., 1992), so alpha-2 agonism may attenuate an established rigidity state while deepening sedation or, under other conditions, further reduce postural support. The direction must therefore be determined from longitudinal change in axial muscle activity, co-contraction and passive resistance at comparable arousal and ventilation rather than from posture alone (§3.5; Table 3).

**TABLE 3:** Predicted findings under each candidate account of the flexed presentation.

Measurement	Generalized sedation	Disproportionate impairment of axial antigravity control	Flexor-predominant rigidity or dystonia	Mixed or fluctuating state
Axial extensor activity (surface electromyography, paraspinal and cervical extensors)	Reduced in proportion to arousal	Reduced beyond what arousal predicts	Not the primary abnormality; may be normal or increased through co-contraction	Alternating, with periods of each pattern
Trunk flexor activity (surface electromyography, abdominal wall)	Reduced in proportion to arousal	Not increased	Increased	Increased intermittently
Co-contraction (flexor and extensor activity concurrently)	Low	Low	Elevated	Variable within a single episode
Passive resistance to movement (neck and trunk; trained assessment only)	Reduced	Reduced	Increased	Variable
Relationship to arousal (recorded arousal measure)	Postural change tracks arousal	Postural change exceeds what arousal predicts	May dissociate from arousal; rigidity and unresponsiveness can co-occur (Streisand et al., 1993)	Coupling varies over time
Relationship to ventilation (rate, depth, regularity)	Tracks overall depression	Dissociable from ventilation	May impair ventilation mechanically (Haouzi and Tubbs, 2022)	Variable
Effect of alpha-2 co-exposure	Deepens sedation and postural change together	Deepens the reduction in extensor activity	Predicted reduction in rigidity-related electromyographic activity and co-contraction while sedation persists, extending the attenuation of rigidity shown in animals (Lewis et al., 2026b; Weinger et al., 1989); direct axial flexor-extensor and passive-resistance measurements remain unavailable	Direction varies with the prevailing motor state
After naloxone, at restored ventilation	Postural recovery broadly tracks recovery of arousal	Residual axial motor impairment after adjustment for arousal and ventilation	Residual increased tone possible, including through mu-opioid-receptor-independent mechanisms (Wei et al., 2026)	Mixed pattern on serial assessment

All entries are predictions generated by the proposed framework unless an individual citation is given, and all comparisons require assessment at comparable arousal and ventilation (§3.5). Wooden chest syndrome does not appear as a column: it is the severe ventilatory expression of fentanyl-associated rigidity (§3.5), and its flexor-predominant form appears here as the third candidate rather than as a contrasting phenotype. Passive resistance should be assessed only by trained personnel and only after trauma and other contraindications have been excluded (§3.1, §5).

The same pharmacological reasoning applies to medetomidine, an alpha-2 agonist with approximately 100-fold higher receptor affinity than xylazine (Schwartz and Clark, 1998). Identified in the United States illegal drug supply by 2021 (Centers for Disease Control and Prevention, 2026) and subsequently in tested samples across several jurisdictions (Schwarz et al., 2024; Sisco and Appley, 2023), it had reached 72% of tested illicit opioid samples in Philadelphia by late 2024 (Huo et al., 2025). Greater affinity may influence the potency or duration of alpha-2-mediated effects but does not predict the direction or magnitude of the postural response. A confirmed-exposure case series found sinus bradycardia predominant and did not examine postural flexion (Murphy et al., 2025). The modifier framework therefore applies to the wider class of veterinary alpha-2 agonists entering the illicit opioid supply.

Animal studies of fentanyl-analogue neurorespiratory toxicity have characterized these effects in detail (Chamoun et al., 2023), and other preclinical work shows that xylazine modifies fentanyl-associated hypoxia, behavioral demand and interoceptive effects, but none measured axial posture or muscle tone; they establish pharmacological interaction, not the direction of motor change (Bender et al., 2025; Choi et al., 2024; Sadek et al., 2024). The direction of the postural effect therefore remains unmeasured.

### 3.5 Competing accounts of the flexed posture

Fentanyl-associated increases in muscle tone extend well beyond the syndrome most often named. Three overlapping levels of expression should be distinguished: generalized skeletal muscle rigidity; involvement of respiratory and upper-airway musculature; and wooden chest syndrome as the clinically severe ventilatory expression of this spectrum. Generalized rigidity involves the limbs and trunk. In rats, fentanyl increases electromyographic activity in the gastrocnemius and the rectus abdominis (Lui et al., 1989) and at overdose doses produces tonic contraction of most skeletal muscles with a measurable rise in oxygen consumption (Haouzi and Tubbs, 2022); in humans, high-dose morphine produces abdominal wall rigidity with reduced thoracic compliance, an effect markedly potentiated by concurrent nitrous oxide (Freund et al., 1973). Where the diaphragm, intercostals and laryngeal musculature are involved the ventilatory consequence narrows (Cavallo et al., 2023; Torralva and Janowsky, 2019), and the mechanical burden of wooden chest syndrome appears to lie substantially in glottic closure rather than chest wall contraction alone (Bennett et al., 1997; Pergolizzi et al., 2021; Scamman, 1983); in a rat model, fentanyl produced rapid and sustained vocal cord closure that naloxone prevented but did not reverse (Miner et al., 2021). Rigidity is common at high anesthetic doses and rapid administration (Grell et al., 1970; Neidhart et al., 1989; Streisand et al., 1993), is also reported during procedural sedation and pain procedures at analgesic doses (Çoruh et al., 2013; McElrath et al., 2024), and has been observed in community overdose (Buxton et al., 2018); across ten repeated high-dose exposures in rats, fentanyl increased electromyographic activity by a mean of 291% with no significant attenuation across exposures (Obert et al., 2025), which bears on a population exposed repeatedly rather than once.

Wooden chest syndrome and the flexed posture cannot therefore be treated as opposite poles of a single tone axis. If opioid exposure can drive contraction of the abdominal wall and other trunk flexors (Freund et al., 1973; Lui et al., 1989), a rigid posture may itself be a flexed one, and the postural abnormalities of the current epidemic may be induced by rigidity of muscle effectors other than the chest wall (Ganos et al., 2025). The community record points the same way: the Vancouver series recorded flexed posturing, clenched hands and abnormal posturing within a rigidity category, with muscle rigidity the single most common atypical presentation in 240 of 1,581 responses (Kinshella et al., 2018); an independent rapid ethnographic study described body and chest rigidity as distinguishing fentanyl-related overdose (Mayer et al., 2018); and events involving rigidity rose from 10.4% to 18.9% as fentanyl entered the supply (Notta et al., 2019). Nor does the appearance of sedation exclude increased tone, since rigidity and unresponsiveness occurred together in volunteers (Streisand et al., 1993). Forward flexion cannot be treated as visual evidence of hypotonia.

The mechanisms remain incompletely resolved. Classical models implicate central noradrenergic and glutamatergic pathways: rat work implicates the locus coeruleus and coerulospinal noradrenergic projection (Lui et al., 1989, 1990), denervation abolishes the response (Lui et al., 1993), the spinal effector appears to be the alpha-1 adrenoceptor (Lui et al., 1995), and coerulospinal glutamatergic transmission also contributes (Fu et al., 1997). More recent work identifies a distinct and potentially complementary mechanism: fentanyl, but not morphine, directly blocks ether-à-go-go class potassium channels in cervical spinal motoneurons, increasing intrinsic excitability and producing a tonic component of muscle activity independently of mu-opioid receptor activation (Wei et al., 2026). Spinal opioid action can also raise tone directly: in rats with mild thoracic spinal cord injury, intrathecal morphine increased hindlimb tone through spinal mu- and delta-opioid receptors (Kawakami et al., 2022). Several mechanisms are implicated and none is established as sufficient. Spinal noradrenergic control is distributed across the locus coeruleus, subcoeruleus and A5 and A7 groups, but tracing and lesion studies differ on their projections and relative contributions to ventral-horn motor control (Clark and Proudfit, 1991; Loughlin et al., 1986a,1986b; Lyons et al., 1989; Westlund et al., 1984). The direction of the fentanyl effect on these systems is likewise state- and region-dependent (Torralva and Janowsky, 2019). Anatomy alone therefore cannot distinguish postural suppression from rigidity; measurement can.

Four accounts of the acute flexed posture therefore remain plausible, and the evidence currently available does not select among them.

*Generalized sedation with loss of postural support* Supervised consumption settings routinely assess graded levels of sedation among people who arrive intoxicated (Wishik et al., 2021), and opioid sedation has its own electrocortical signature (Montandon and Horner, 2019; Montandon et al., 2016). On this account, postural change accompanies sedation and no separate postural mechanism is required. The objection that muscle atonia in comparable states involves the lower limbs and produces falls (Ganos et al., 2025) has force against a chronic ambulant presentation but not against the acute seated state delimited in §3.1.

*Disproportionate impairment of axial antigravity control* Axial extensor output is reduced beyond what arousal predicts at comparable ventilation, without increased flexor co-contraction or passive resistance (§§3.2–3.3).

*Flexor-predominant rigidity or dystonia* Sustained or fluctuating contraction of trunk flexors producing a hypertonic rather than hypotonic posture, consistent with the rigidity literature above.

*A mixed or fluctuating motor state* More than one of the above, which may be the most clinically realistic; the community record describes flexion and rigidity in the same individuals (Kinshella et al., 2018).

Basal-ganglia and dopaminergic mechanisms remain exploratory. Ganos and colleagues propose dopaminergic disruption linking these postures to camptocormia, and focal lenticular lesions can produce acute dystonic camptocormia (Ganos et al., 2025; Nieves et al., 2001); preclinically, xylazine induces dopamine release and augments fentanyl-associated effects (Trinko et al., 2024), sustained fentanyl exposure alters striatal neuronal activity independently of opioid receptors (Yarotskyy et al., 2024), and acute fentanyl-xylazine co-exposure alters the excitability of dopamine type 2 receptor-expressing striatal medium spiny neurons (Yarotskyy et al., 2026). None establishes a postural mechanism for the acute presentation.

Persistence of flexion and altered consciousness after naloxone does not discriminate among these accounts. Prolonged sedation after naloxone is described in medetomidine-involved overdose (Centers for Disease Control and Prevention, 2026), but adequate ventilation may recover before posture or arousal, and residual flexion is compatible with incompletely antagonized or recurrent opioid action, co-intoxicant sedation, hypoxic or hypercapnic contribution, or a mu-opioid-receptor-independent motor mechanism (Love et al., 2026b; Wei et al., 2026); severe hypoxemia has also been proposed to shift the pathophysiology from opioid-dependent to hypoxia-dependent (Lewis et al., 2026a). Even where an alpha-2 component is confirmed, what has been distinguished is opioid sedation from alpha-2 sedation, not flexion from sedation. It is therefore recorded as an outcome rather than interpreted, and its clinical implications are addressed in §4.2.

The accounts differ in what they predict about muscle activity, not appearance. The central requirement is that axial tone be assessed against arousal at comparable ventilation; Table 3 sets out the finding predicted under each account for each measurement. Concurrent surface electromyography of paraspinal and abdominal groups, standardized assessment of passive resistance (Ganguly et al., 2021), a recorded arousal measure and ventilatory monitoring would permit prospective comparison of the accounts. Inertial measurement units measure trunk and neck flexion to within approximately 1.6 and 2.9 degrees respectively in a protocol-specific validation study (Morrow et al., 2017), and chest-worn respiratory sensors are being evaluated in a supervised injecting setting (Tas et al., 2024).

Three testable predictions follow.

*Prediction one* At comparable arousal and ventilation, disproportionately reduced axial extensor activity would favor the antigravity account; extensor activity that tracks arousal would favor generalized sedation.

*Prediction two* The direction of the alpha-2 effect would help distinguish the antigravity and rigidity accounts. Further reduction in axial extensor activity would favor impaired antigravity control, whereas reduced flexor activity, co-contraction and passive resistance despite persistent sedation would favor rigidity modulation, extending the pattern shown for respiratory-muscle rigidity in animals (Haouzi and Tubbs, 2022).

*Prediction three* After naloxone restores ventilation, postural recovery that tracks recovery of arousal would favor generalized sedation; residual axial motor impairment after adjustment for arousal and ventilation would favor disproportionate antigravity dysfunction.

## 4 Implications for clinical recognition, naloxone strategy, and harm reduction practice

Although the motor state underlying the flexed presentation remains unresolved, several practical implications are shared across the accounts considered in §3.5. Whether the posture reflects generalized sedation, disproportionate impairment of antigravity control, flexor-predominant rigidity or a mixed state, it is not fully described by the classical overdose triad. Postural appearance should prompt assessment for toxicity, but urgency should be determined by ventilation and other signs of physiological compromise rather than by posture, and appearance is insufficient to establish the underlying tone state or mechanism at the point of response. These priorities hold while the mechanism is unresolved, though subsequent assessment and interpretation may differ according to the motor state involved.

### 4.1 Recognition

Bystander, peer and clinician recognition of opioid overdose has historically been organized around the classical triad of depressed consciousness, depressed respiration and miosis. Brief training enables bystanders to distinguish opioid overdose from non-opioid emergencies in scenario-based assessment, but that work was developed in the heroin and prescription-opioid era and presumes a typical presentation (Green et al., 2008). The flexed presentation may not match the depressed-consciousness and depressed-breathing presentation emphasized in conventional overdose-recognition training (Green et al., 2008). A person sustained in forward flexion with episodic recovery of upright posture may appear heavily sedated rather than overdosing, particularly where respiratory depression is incomplete or the observer’s reference frame is the unresponsive presentation that overdose education has emphasized. The window for intervention is therefore at risk not because bystanders fail to act on what they see, but because what they see is not yet recognized as overdose.

**FIGURE 3:**
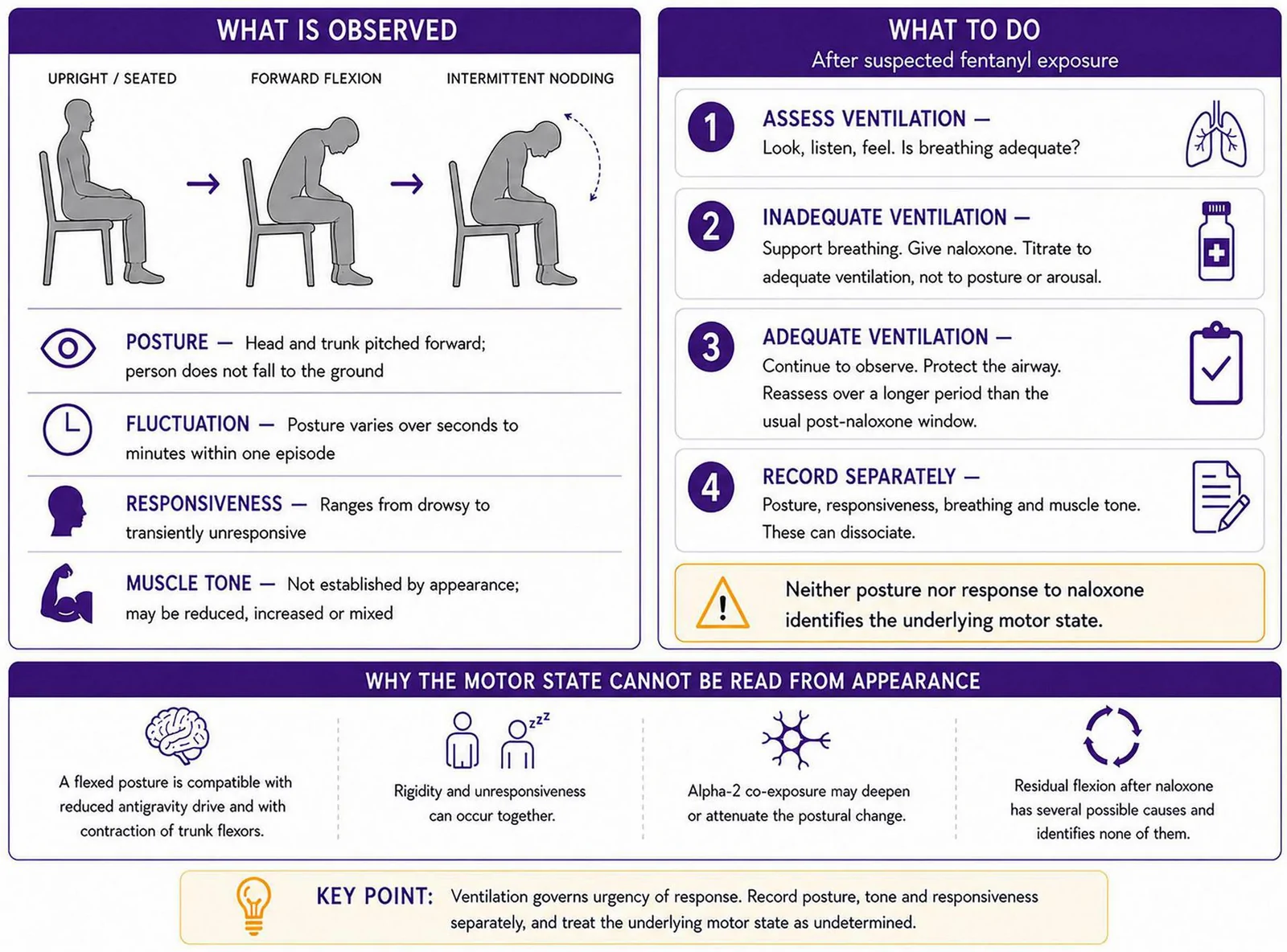
Recognizing the flexed presentation after suspected fentanyl exposure. The left panel shows the observed features of the presentation, including the fluctuating posture and the fact that muscle tone is not established by appearance. The right panel sets out a ventilation-first response sequence, with posture, responsiveness, breathing and muscle tone recorded separately. The lower band summarizes why the underlying motor state cannot be read from appearance or from response to naloxone.

The bedside task is narrower than it may appear. It is not to determine which mechanism is operating, since that requires measurement of muscle activity and tone against arousal (§3.5), but to recognize possible drug toxicity, assess ventilation, and avoid inferring mechanism from appearance (Figure 3). Five considerations guide this. First, fluctuation is characteristic, and transient improvement should not be taken to establish resolution. Second, in fentanyl-dominant settings, sustained semi-upright flexion after suspected opioid use should prompt assessment for possible drug toxicity rather than dismissal as volitional rest. Third, postural and respiratory state can dissociate: slow, shallow or irregular breathing shifts interpretation toward overdose regardless of posture (Montandon and Slutsky, 2019; Pattinson, 2008). Fourth, the fentanyl-era motor presentation is variable and includes rigidity, dyskinesia and other abnormal posturing (Buxton et al., 2018; Kinshella et al., 2018; Notta et al., 2019); in an appropriate exposure context, atypical motor features should prompt consideration of fentanyl-era pharmacology, while neurological and traumatic causes remain in the differential. Fifth, naloxone response is not diagnostic. Alpha-2 effects are not reversed by naloxone (Gupta et al., 2023), and partial improvement with persistent flexion is also compatible with incompletely antagonized opioid action, other co-intoxicant sedation and hypoxic contribution, and identifies none of them. It should not by itself be read as failed reversal or justify repeat naloxone while ventilation is adequate (Fairbairn et al., 2017).

### 4.2 Naloxone strategy in polysubstance contexts

Naloxone reverses opioid effects mediated at the mu-opioid receptor. It does not reverse alpha-2 adrenergic agonism, nor would it be expected to reverse a motor component arising through a mu-opioid-receptor-independent mechanism, as recently described experimentally for fentanyl-associated rigidity (Wei et al., 2026). Naloxone response is therefore an unreliable guide to the mechanism of increased muscle tone: fentanyl-associated rigidity has been reversed by naloxone in some reports (Çoruh et al., 2013) and has persisted despite naloxone in others, with naloxone preventing but not reversing fentanyl-induced vocal cord closure in a rat model (Miner et al., 2021). The clinical endpoint that should govern naloxone dosing is therefore restoration of adequate ventilation, not normalization of posture or arousal.

Persistent forward flexion or altered consciousness despite adequate ventilation does not, by itself, establish inadequate naloxone dosing or failed reversal, nor should it be attributed to a particular co-exposure. Restoration of adequate ventilation is not equivalent to complete reversal of all opioid effects, and the residual state is compatible with incompletely antagonized opioid action, recurrent toxicity as naloxone is cleared, sedation from an alpha-2 agonist or other co-intoxicant (Gupta et al., 2023), and hypoxic or hypercapnic contribution. A co-exposure need not be sedative to matter: in rats, naloxone was less potent and less effective at reversing cardiorespiratory depression from a fentanyl-methamphetamine mixture than from fentanyl alone, recovering baseline oxygen saturation in a smaller proportion of animals and requiring substantially longer to do so (Vazquez et al., 2025). Their relative contributions cannot be assigned confidently at the bedside, and clinical appearance does not reliably identify alpha-2 exposure (Love et al., 2026b); in a pilot cohort with toxicological confirmation, pre-hospital naloxone doses were similar whether or not xylazine was detected (Merchant et al., 2026). Immediate naloxone titration does not require distinguishing among these possibilities; it should remain guided by ventilation.

Continued observation, airway positioning, ventilatory support when required, and repeated monitoring of ventilation and oxygenation are appropriate because recurrent opioid toxicity and prolonged co-intoxicant effects may occur. Supportive care remains central when alpha-2 co-exposure is suspected, consistent with current consensus guidance (Perrone et al., 2024); a community overdose response team managing xylazine-involved overdoses similarly centered oxygenation rather than consciousness in naloxone titration (Pacheco Bufanda et al., 2025). Conversely, persistent hypoventilation after initial naloxone does require repeat dosing with airway and ventilatory support, because inadequate ventilation, not residual flexion, is the threat to life. Escalating naloxone solely for persistent flexion or altered consciousness despite adequate ventilation risks precipitating withdrawal without addressing the residual sedative load (Fairbairn et al., 2017; Gupta et al., 2023).

### 4.3 Harm reduction practice and stigma

Harm reduction services and supervised consumption sites have first-hand experience of the spectrum described here and contributed early formal descriptions of atypical fentanyl-era presentations (Kinshella et al., 2018). Translating this framework into peer education and overdose-response protocols requires acknowledging that fentanyl-era presentations differ from those on which heroin-era training was built. Postural flexion and nodding may warrant inclusion alongside the classical triad in recognition training, with attention to the fluctuating and partially reversible character of the state. The framework also bears on stigma. Nodding may be interpreted as evidence of chosen intoxication rather than as a possible manifestation of pharmacological toxicity. The same structure of error has been described in correctional opioid use disorder assessment, where clinical variability is read as unreliability rather than as genuine variation in state (Albert, 2026). Addiction stigma is sustained partly by the belief that drug use reflects moral failing rather than a medical condition, with consequences for public attitudes and policy (McGinty and Barry, 2020). Locating the posture in brainstem and spinal motor physiology places it among the pharmacological effects of exposure rather than among the choices of the person exposed.

Two operational possibilities follow. Overdose-recognition curricula have traditionally emphasized depressed consciousness and depressed breathing (Green et al., 2008); upright and semi-upright presentations with sustained forward flexion could be added, together with the implications for naloxone strategy in suspected polysubstance exposure. Standardized behavioral descriptors could also be incorporated into supervised consumption site documentation alongside existing event-level overdose coding, providing a low-cost foundation for the prospective observational research the framework requires and aligning with wearable-sensor methods now being piloted in supervised injecting settings (Tas et al., 2024).

## 5 Future directions

Three linked studies follow, in order of increasing methodological complexity.

*Prospective characterization* Observational work in supervised consumption and outreach settings could establish how often the presentation occurs, how it is distributed across exposure contexts, and how reliably trained observers apply the descriptors in §3.1. A minimum dataset would capture posture, responsiveness, respiratory rate, depth and regularity, naloxone timing and dose, and an explicit tone field recorded as reduced, increased, mixed or indeterminate. Formal tone assessment should be attempted only by trained personnel and only where trauma and other contraindications have been excluded; otherwise indeterminate is the appropriate entry. Time-stamped video may supplement these observations where an ethically approved consent framework permits. The critical change is that tone is recorded separately from posture.

*Discriminating physiology* Separating the candidate accounts requires axial muscle activity and passive resistance to movement to be assessed against arousal at comparable ventilation (§3.5). This calls for surface electromyography of paraspinal and abdominal groups, standardized assessment of passive resistance, a validated arousal measure and concurrent ventilatory monitoring, alongside inertial measurement of trunk and neck flexion (Morrow et al., 2017) and chest-worn respiratory sensing (Tas et al., 2024). Kinematics alone will not answer the question, since posture does not establish tone. The relevant instruments are available, but they have not been integrated or validated in this setting. The principal challenge is developing an ethically and operationally feasible protocol for their concurrent use during an acute exposure episode.

*Exposure-stratified analysis of alpha-2 modification* Comparative studies could determine whether alpha-2 co-exposure shifts neuromuscular activity, in which direction, and under what conditions. Fentanyl-dominant, fentanyl-plus-xylazine and fentanyl-plus-medetomidine exposures would be compared using drug checking of the consumed sample where available (Karamouzian et al., 2018; Tupper et al., 2018) together with confirmatory biological toxicology, which is necessary because clinical presentation alone identifies alpha-2 exposure poorly (Love et al., 2026b). The primary outcomes would be axial muscle activity and passive resistance rather than posture, with kinematics as a complementary measure.

One line of inquiry warrants explicit caution. Because alpha-2 antagonists reverse alpha-2 agonist effects, they may appear an intuitive intervention where alpha-2 co-exposure is suspected, and clinical investigation of atipamezole in human alpha-2 agonist toxicity has been advocated on that basis (Blumenberg and Murphy, 2026). Atipamezole, however, augmented opioid-induced rigidity and produced a small increase in baseline tonic electromyographic activity in rats (Weinger and Bednarczyk, 1994), and cannot be presumed to correct a motor sign of uncertain origin. Investigation of alpha-2 antagonism should therefore be confined to controlled research settings, and nothing in this framework supports its use in community overdose response. The clinical recommendations in §4 do not depend on completion of this work; these studies concern mechanism, not immediate management.

## 6 Limitations

Several boundaries define what this synthesis can and cannot establish. Foremost, the motor state underlying the flexed presentation has not been measured directly. Rigidity, consciousness and ventilation have been measured concurrently in anesthetic settings (Streisand et al., 1993), but not with the distinction between axial extensor and flexor activity that the candidate accounts require, and not during exposure to illicitly manufactured fentanyl. The four accounts considered in §3.5 therefore remain empirically unresolved, and the proposed disproportionate impairment of axial antigravity control should be understood as a primary hypothesis rather than an established mechanism. The contribution of the framework is to specify the measurements that would distinguish this account from generalized sedation, flexor-predominant rigidity and mixed motor states, rather than to infer mechanism from posture alone.

The phenotype itself also requires prospective validation. Its frequency, boundaries and reproducibility are unknown, and the operational description in §3.1 has not been tested for inter-rater reliability or diagnostic performance. It is therefore a research definition intended to support systematic observation, not a validated diagnostic category. The predictions in §3.5 are similarly prospective. In addition, the synthesis prioritizes fentanyl and the alpha-2 agonists xylazine and medetomidine. Other common co-exposures, including alcohol, benzodiazepines, gabapentinoids and stimulants, may independently alter arousal, muscle tone and motor expression. Benzodiazepine pretreatment, for example, may attenuate fentanyl-associated thoracic rigidity under anesthetic conditions (Neidhart et al., 1989), illustrating how the observed presentation may vary with drug-supply composition. Such combinations are common: in the 2023 Canadian Substance Use Survey, polysubstance use was widespread and alcohol-opioid and opioid-stimulant combinations carried elevated self-reported harm (Albert and Arthur, 2026b).

The evidence is also concentrated in North American fentanyl-dominant settings, particularly supervised consumption services and jurisdictions affected by alpha-2 adulterants. This gives the framework direct relevance to those settings but leaves its transferability to other regional drug supplies uncertain. Fatal drug-harm trajectories diverge substantially across high-income settings (Albert and Arthur, 2026a), so the epidemiological context in which this presentation is encountered is not uniform. The literatures brought together here, spanning community observation, anesthesia, toxicology, postural neurophysiology, animal pharmacology and surveillance, also differ substantially in populations, exposures and outcomes. Their convergence supports mechanistic plausibility, but it cannot substitute for direct causal evidence in people experiencing the acute presentation. These differences, including areas of disagreement, are made explicit in Table 1.

Structured narrative synthesis with mechanistic bridging is appropriate to a question whose evidence is fragmented across otherwise separate fields, but it necessarily involves judgment in source selection, evidentiary weighting and inference across non-equivalent models. The search strategy, evidence hierarchy and inferential boundaries governing that judgment are set out in §2 and Supplementary Materials 1 and 2. The resulting framework should therefore be read as a testable map of the candidate mechanisms and the observations needed to distinguish them, not as a validated phenotype or a settled account of its neurophysiology. None of these limitations bears on the clinical priorities set out in §4, which hold across the candidate accounts.

## 7 Conclusion

The flexed posture described in fentanyl-era intoxication and overdose reports presents a deceptively simple clinical observation. Its outward appearance is familiar, but its underlying motor state is not. Forward flexion may accompany generalized sedation, disproportionate impairment of axial antigravity control, flexor-predominant rigidity or a combination of these processes. The central problem is therefore not whether the posture can be seen, but whether appearance alone can reveal the physiology producing it. The evidence reviewed here gives no basis for supposing that it can.

This framework brings together postural neurophysiology, opioid pharmacology, rigidity research and observations from supervised consumption settings to define that uncertainty more precisely. Upright posture depends on continuously regulated descending motor drive; fentanyl can affect respiratory, arousal and motor systems through partially dissociable pathways; and alpha-2 adulterants may modify the resulting presentation differently according to the prevailing motor state. On this basis, disproportionate impairment of axial antigravity control is advanced as a primary, testable hypothesis rather than as a settled explanation. The competing accounts make different predictions about axial extensor activity, flexor co-contraction and passive resistance when assessed against arousal at comparable ventilation. The framework therefore shifts the inquiry from naming the posture to measuring the motor state beneath it.

That distinction also matters clinically. A person who remains seated or standing in sustained forward flexion may not match the depressed-consciousness and depressed-breathing presentation emphasized in conventional overdose-recognition training. The posture should prompt assessment for drug toxicity, but it should not be used to infer respiratory severity, muscle tone, co-exposure or likely response to naloxone. Ventilation and physiological compromise remain the immediate determinants of urgency, while persistent flexion or altered consciousness after restoration of breathing requires observation and supportive care rather than mechanistic attribution from appearance alone (§4).

The broader lesson is that respiratory depression, arousal and motor control need not change in parallel, and visual similarity need not imply physiological equivalence. Resolving these relationships will require prospective observation, neuromuscular measurement and exposure-confirmed studies. The task now is to replace visual inference with physiological evidence.

## Data Availability

The original contributions presented in the study are included in the article/Supplementary material, further inquiries can be directed to the corresponding author.
