# Supplementary material for: The opioid flexion phenotype: postural flexion and nodding in the fentanyl era
Source: Front Neurosci. 2026 Sep 10;20:1910436. doi: 10.3389/fnins.2026.1910436 (PMC13601219; doi:10.3389/fnins.2026.1910436)
Supplement: Supplementary file 1 [file Table_1.docx]

**Supplementary Material 1. Database search strategy**

Supplement to: The Opioid Flexion Phenotype: Postural Flexion and Nodding in the Fentanyl Era

**Overview**

This narrative review searched PubMed (NLM), Embase (Elsevier), and PsycINFO (APA) for English-language literature published from database inception to July 2026. The search was structured around five thematic clusters reflecting the review's analytical scope: (1) postural control and reticulospinal neurophysiology; (2) mu-opioid receptor pharmacology, fentanyl, and brainstem respiratory and motor control, including opioid action at motoneurons; (3) xylazine, medetomidine, and other alpha-2 adrenergic agonists in the illicit drug supply; (4) observational studies of fentanyl-era overdose presentations including atypical motor phenomena and supervised consumption site surveillance data; and (5) drug-induced movement disorders and axial postural abnormality. Selected foundational publications from earlier decades were retained where they remained the canonical references for posture neurophysiology and reticulospinal organisation. Reference lists of major reviews, public health surveillance reports, and clinical case literature on fentanyl-associated rigidity were hand-searched for additional sources (Greenhalgh and Peacock, 2005). Database selection was tailored to each cluster according to the literature concerned, so that the databases indexing the relevant field were searched for that cluster rather than all three databases being applied uniformly across clusters. Colloquial and descriptive terms for the presentation, including fentanyl nod, fentanyl fold and heroin hunch, were included as free-text terms in Cluster 4 alongside the formal descriptors, because these terms appear in the community and clinical literature in which the presentation is described.

**Cluster 1. Postural control and reticulospinal neurophysiology**

***PubMed search string***

("postural control"[Title/Abstract] OR "postural tone"[Title/Abstract] OR "axial tone"[Title/Abstract] OR "reticulospinal"[Title/Abstract] OR "reticulospinal tract"[Title/Abstract] OR "vestibulospinal"[Title/Abstract] OR "locus coeruleus"[Title/Abstract]) AND ("brainstem"[Title/Abstract] OR "pontomedullary"[Title/Abstract] OR "muscle tone"[Title/Abstract] OR "axial musculature"[Title/Abstract] OR "trunk flexion"[Title/Abstract] OR "head drop"[Title/Abstract] OR "neck flexion"[Title/Abstract])

***Embase adaptation***

('postural control'/exp OR 'postural tone' OR 'reticulospinal tract'/exp OR 'locus coeruleus'/exp) AND ('brain stem'/exp OR 'pontomedullary' OR 'muscle tone'/exp OR 'axial musculature' OR 'trunk flexion')

**Cluster 2. Mu-opioid receptor pharmacology, fentanyl, and brainstem respiratory and motor control**

***PubMed search string***

("fentanyl"[MeSH] OR "fentanyl"[Title/Abstract] OR "synthetic opioid"[Title/Abstract] OR "illicitly manufactured fentanyl"[Title/Abstract]) AND ("respiratory depression"[Title/Abstract] OR "opioid-induced respiratory depression"[Title/Abstract] OR "respiratory rhythm"[Title/Abstract] OR "preBötzinger"[Title/Abstract] OR "Kölliker-Fuse"[Title/Abstract] OR "wooden chest"[Title/Abstract] OR "muscle rigidity"[Title/Abstract] OR "thoracic rigidity"[Title/Abstract] OR "motoneuron"[Title/Abstract] OR "motor neuron"[Title/Abstract] OR "electromyography"[Title/Abstract] OR "muscle tone"[Title/Abstract] OR "potassium channel"[Title/Abstract])

***Embase adaptation***

('fentanyl'/exp OR 'synthetic opioid') AND ('respiratory depression'/exp OR 'opioid induced respiratory depression' OR 'pre-Botzinger complex'/exp OR 'wooden chest' OR 'muscle rigidity'/exp OR 'thoracic rigidity' OR 'motoneuron'/exp OR 'electromyography'/exp OR 'muscle tone'/exp)

**Cluster 3. Xylazine, medetomidine, and alpha-2 adrenergic agonists in the illicit drug supply**

***PubMed search string***

("xylazine"[MeSH] OR "xylazine"[Title/Abstract] OR "medetomidine"[Title/Abstract] OR "dexmedetomidine"[Title/Abstract] OR "detomidine"[Title/Abstract] OR "alpha-2 adrenergic"[Title/Abstract] OR "alpha-2 agonist"[Title/Abstract]) AND ("illicit drugs"[Title/Abstract] OR "drug supply"[Title/Abstract] OR "fentanyl adulteration"[Title/Abstract] OR "drug overdose"[MeSH] OR "overdose"[Title/Abstract] OR "polysubstance"[Title/Abstract] OR "drug checking"[Title/Abstract])

***Embase adaptation***

('xylazine'/exp OR 'medetomidine'/exp OR 'alpha-2 adrenergic receptor agonist'/exp) AND ('illicit drug'/exp OR 'drug supply' OR 'drug adulteration'/exp OR 'drug overdose'/exp OR 'drug checking')

**Cluster 4. Fentanyl-era overdose presentations and supervised consumption site surveillance**

***PubMed search string***

("supervised injection"[Title/Abstract] OR "supervised consumption"[Title/Abstract] OR "safe injection facility"[Title/Abstract] OR "drug consumption room"[Title/Abstract] OR "harm reduction"[MeSH]) AND ("overdose"[Title/Abstract] OR "drug overdose"[MeSH] OR "naloxone"[MeSH] OR "atypical presentation"[Title/Abstract] OR "rigidity"[Title/Abstract] OR "dyskinesia"[Title/Abstract] OR "postural"[Title/Abstract] OR "nodding"[Title/Abstract] OR "fentanyl nod"[Title/Abstract] OR "fentanyl fold"[Title/Abstract] OR "heroin hunch"[Title/Abstract] OR "forward flexion"[Title/Abstract] OR "truncal flexion"[Title/Abstract])

***PsycINFO adaptation***

(supervised injection OR supervised consumption OR harm reduction) AND (overdose OR naloxone OR atypical presentation OR postural OR nodding OR "fentanyl nod" OR "fentanyl fold" OR "heroin hunch")

**Cluster 5. Drug-induced movement disorders and axial postural abnormality**

***PubMed search string***

("camptocormia"[Title/Abstract] OR "axial postural abnormality"[Title/Abstract] OR "dystonia"[MeSH] OR "drug-induced movement disorder"[Title/Abstract] OR "movement disorders"[MeSH] OR "basal ganglia"[Title/Abstract] OR "bent spine"[Title/Abstract] OR "dropped head"[Title/Abstract]) AND ("opioid"[Title/Abstract] OR "fentanyl"[Title/Abstract] OR "xylazine"[Title/Abstract] OR "substance-related disorders"[MeSH] OR "posture"[MeSH])

***Embase adaptation***

('camptocormia'/exp OR 'dystonia'/exp OR 'drug induced movement disorder' OR 'movement disorder'/exp OR 'basal ganglion'/exp) AND ('opiate'/exp OR 'fentanyl'/exp OR 'xylazine'/exp OR 'posture'/exp)

***PsycINFO adaptation***

(camptocormia OR dystonia OR "movement disorder" OR "basal ganglia") AND (opioid OR fentanyl OR xylazine OR posture)

**Hand-searching**

Reference lists of the following sources were hand-searched: (1) the observational report of atypical overdose presentations at the Vancouver supervised injection site (Kinshella et al. 2018) and the rapid ethnographic study from the same setting (Mayer et al. 2018); (2) canonical reviews of postural control and reticulospinal organisation (Takakusaki 2017; Takakusaki et al. 2004, 2016; Deliagina et al. 2006; Cacciatore et al. 2024); (3) reviews of fentanyl-associated muscle rigidity and wooden chest syndrome (Torralva and Janowsky 2019; Pergolizzi et al. 2021); (4) the movement-disorder account of the presentation (Ganos et al. 2025); (5) surveillance reports and peer-reviewed detection studies on xylazine and medetomidine (Friedman et al. 2022; Kariisa et al. 2023; Schwarz et al. 2024; Huo et al. 2025); and (6) clinical case literature on fentanyl-induced rigidity in community and procedural settings (Buxton et al. 2018; Çoruh et al. 2013; McElrath et al. 2024). Forward citation-tracking was conducted from the foundational neurophysiological papers (Peterson et al. 1979; Gurfinkel et al. 2006; Horak 2006) and from the primary rigidity literature (Lui et al. 1989; Weinger et al. 1989) using Web of Science and Google Scholar. Reference and citation tracking from the primary opioid-rigidity literature was also used to identify experimental studies of alpha-2 agonism and antagonism in opioid-associated motor rigidity.

**Eligibility summary**

| **Criterion** | **Specification** |
| --- | --- |
| Language | English |
| Date range | Database inception to July 2026 |
| Publication type | Original research articles, narrative and systematic reviews, public health surveillance reports and agency advisories, clinical case reports, anaesthesia case literature, and primary neurophysiological and pharmacological studies |
| Excluded | Conference abstracts and meeting proceedings without full peer-reviewed publication; preprints; non-peer-reviewed grey literature except CDC and equivalent public health agency reports; opinion pieces without empirical or mechanistic content |
| Special inclusions | Foundational pre-database-era references retained where canonical for posture neurophysiology and reticulospinal organisation |
