# Supplementary material for: The opioid flexion phenotype: postural flexion and nodding in the fentanyl era
Source: Front Neurosci. 2026 Sep 10;20:1910436. doi: 10.3389/fnins.2026.1910436 (PMC13601219; doi:10.3389/fnins.2026.1910436)
Supplement: Supplementary file 2 [file Table_2.docx]

**Supplementary Material 2. Source selection criteria and evidence hierarchy**

Supplement to: The Opioid Flexion Phenotype: Postural Flexion and Nodding in the Fentanyl Era

**Overview**

Sources retrieved through the search strategy outlined in Supplementary Material 1 were selected and prioritised according to the explicit hierarchy below. Different evidence types were given primacy for different kinds of claim, reflecting the heterogeneity of the literatures the review bridges. Two principles govern the use of animal-model data. Where such data are offered in support of a claim about the human presentation, they establish mechanistic plausibility only and are identified as such in the text. Where they function instead as evidence for or against a candidate account of the underlying motor state, they are reported at full weight and in whichever direction they point. Where reports diverge, the divergence is documented rather than resolved, and both directions of evidence are retained.

**Evidence hierarchy by claim type**

| **Claim type** | **Primary evidence tier** | **Secondary evidence tier** | **Excluded for this claim type** |
| --- | --- | --- | --- |
| Clinical claims about the fentanyl-era presentation | Human observational data from supervised consumption and outreach settings; rapid ethnographic study; prospective clinical cohorts with toxicological confirmation; medical examiner data | Clinical case reports of community-context presentations; public health surveillance reports and agency advisories | Animal-model data presented as direct evidence of the human presentation; media reports |
| Mechanistic claims about axial postural tone and reticulospinal organisation | Foundational neurophysiological reviews and primary studies; established posture-control literature; primary animal electrophysiology of reticulospinal organisation | Functional neuroimaging; comparative animal studies where convergent with human data | Single-author opinion pieces; non-peer-reviewed sources |
| Mechanistic claims about mu-opioid receptor pharmacology and brainstem respiratory control | Primary respiratory-control literature including targeted receptor deletion and site-specific pharmacology; pharmacology reviews of MOR distribution and signalling | Human electrocortical studies of opioid sedation; animal-model studies of fentanyl analogue neurorespiratory toxicity | Respiratory or oxygenation endpoints presented as evidence of motor or postural output |
| Mechanistic claims about opioid action at spinal and brainstem motor pools | Primary electrophysiology and receptor mapping in spinal and brainstem motor circuits, including intrathecal and site-specific pharmacology, reported in whichever direction the findings point | Behavioural pharmacology where motor output is directly recorded; comparative animal studies of motoneuron excitability | Inference from respiratory motor pools to axial postural pools presented as established rather than analogical |
| Claims about fentanyl-associated muscle rigidity | Primary animal electromyography and lesion or antagonist studies of opioid-induced rigidity; human volunteer studies with quantified incidence; primary channel and receptor physiology | Anaesthesia case literature; community-context case reports; mechanistic pharmacology reviews of opioid-induced rigidity | Wooden chest syndrome treated as the whole of fentanyl-associated rigidity; single-case anecdotes without mechanistic discussion |
| Claims about alpha-2 adulterant prevalence | CDC and equivalent agency surveillance; primary forensic detection studies; peer-reviewed surveillance research | Public health agency advisories; clinical case series with analytical confirmation | Media reports without supporting peer-reviewed source |
| Claims about alpha-2 modification of motor or postural state | Controlled animal pharmacology with recorded motor or electromyographic outcomes, reported in whichever direction the findings point; prospective human cohorts with toxicological confirmation | Behavioural pharmacology of fentanyl and alpha-2 co-exposure; neurorespiratory endpoints identified as such | Neurorespiratory or brain-oxygenation endpoints presented as postural evidence; inference of exposure from clinical appearance alone |
| Claims about overdose recognition and naloxone strategy | Prospective clinical cohorts with toxicological confirmation; observational cohorts of naloxone administration in fentanyl-era settings; empirical evaluations of recognition training | Clinical practice guidelines; expert consensus documents in harm reduction practice; community response case series | Heroin-era recognition data presented without consideration of fentanyl-era context; attribution of residual clinical state to a specific co-exposure without analytical confirmation |
| Methodological claims about narrative review and postural measurement | Established narrative review methodology guidance; validated instrumentation studies with reported error characteristics; validated point-of-care drug checking studies | Methodology overviews and review typologies; consensus nosology for axial postural abnormality | Validation studies applied beyond the protocol and population in which they were performed |
| Claims about drug-induced movement disorders and chronic axial postural abnormality | Clinical descriptions of drug-associated movement disorders in identified cohorts; consensus nosology and cut-off values for axial postural abnormality; primary lesion studies with anatomical localisation | Preclinical studies of striatal and dopaminergic effects of opioid and alpha-2 exposure; case reports of acute dystonic presentations with imaging correlation | Chronic or post-cessation postural abnormality presented as evidence of the acute presentation; preclinical dopaminergic findings presented as an established postural mechanism |

**Application of the hierarchy**

In practice, the hierarchy operated as follows. For each substantive claim, sources were considered in the primary tier first; the secondary tier was used for converging support where primary evidence was available, and as primary support only where the primary tier was empty. The exclusions named in the right-hand column were not used to support claims of the corresponding type. Two applications warrant specific comment. First, animal-model studies of fentanyl analogue neurorespiratory toxicity and of fentanyl-xylazine co-exposure appear in support of mechanistic plausibility and of the alpha-2 modifier argument, and are explicitly distinguished from human observational findings; where such a study measured brain oxygenation rather than posture or muscle activity, this is stated where the study is cited. Second, primary spinal and brainstem motor physiology is drawn on in both directions: studies showing that opioid action increases muscle tone stand alongside those showing reduced excitatory drive to motoneurons, and the resulting divergence is retained. Anaesthesia case literature characterises the ventilatory expression of fentanyl-associated rigidity; the mechanism is not extended unchanged to community-context presentations.

**Recency weighting**

Surveillance and observational data on the contemporary illicit drug supply were weighted toward more recent publications because the supply itself is changing on a timescale of months. Alpha-2 adulterant prevalence claims rely on agency surveillance and peer-reviewed detection studies published from 2022 onward, with earlier sources retained where they document the first appearance of a substance in the illicit supply, reflecting medetomidine's emergence after xylazine's. Clinical claims about alpha-2 co-exposure rely primarily on prospective cohorts with toxicological confirmation, in preference to earlier case-based accounts. Observational data on supervised consumption presentations rely on the available longitudinal and ethnographic record, which predates the widespread appearance of alpha-2 adulterants in the supply; this is a recognised limitation of the observational record. Foundational neurophysiological claims draw on canonical literature regardless of publication date, because those studies remain the principal experimental basis for the anatomy and physiology concerned. Where a foundational account is contested or has been extended by more recent work, both are cited and the disagreement is retained rather than resolved in favour of either. Primary pharmacological literature on opioid-induced rigidity dating from the 1970s to the 1990s is retained on the same basis, as the principal experimental record of that phenomenon.
