# Supplementary material for: The opioid flexion phenotype: postural flexion and nodding in the fentanyl era
Source: Front Neurosci. 2026 Sep 10;20:1910436. doi: 10.3389/fnins.2026.1910436 (PMC13601219; doi:10.3389/fnins.2026.1910436)
Supplement: Supplementary file 3 [file Table_3.docx]

**Supplementary Material 3. Narrative review reporting checklist mapped to SANRA domains**

Supplement to: The Opioid Flexion Phenotype: Postural Flexion and Nodding in the Fentanyl Era

**Scale and application**

The Scale for the Assessment of Narrative Review Articles (SANRA; Baethge, Goldbeck-Wood and Mertens, 2019) comprises six items, each scored from 0 to 2, with a maximum total score of 12. The scale assesses the importance of the review topic, clarity of the aims, description of the literature search, referencing, scientific reasoning, and presentation of relevant evidence. We applied SANRA to the present review, and the item-by-item assessment below shows how each domain is addressed in the manuscript.

**Item-by-item self-assessment**

| **#** | **SANRA item** | **Score** | **Justification** |
| --- | --- | --- | --- |
| 1 | Justification of the article's importance for the readership | 2 | The Introduction establishes the clinical and public-health rationale: the fentanyl era has changed how opioid overdose presents; the forward-flexed posture is repeatedly described but its underlying motor state has not been measured; and existing accounts variously subsume it under sedation, record it within a rigidity framing, or treat it as a drug-induced movement disorder. The §1 discussion of contemporary supply pharmacology and polysubstance adulteration frames why the question is timely for a translational neuroscience readership. |
| 2 | Statement of concrete aims or formulation of questions | 2 | The Introduction states the aim explicitly: to separate the observation from the inference and from the proposed mechanism, to set out the candidate accounts consistent with available evidence, and to specify the measurements that would distinguish them. The primary mechanistic hypothesis is stated, alongside the alternatives against which it is examined. Three falsifiable predictions are formulated, the predicted findings under each candidate account are tabulated, and three studies with candidate methods are specified. |
| 3 | Description of the literature search | 2 | Methods §2 describes the databases searched (PubMed, Embase, PsycINFO), the date range (database inception to July 2026), five thematic clusters, and the hand-searching and forward citation-tracking strategy. Full search strings for all five clusters are provided in Supplementary Material 1. |
| 4 | Referencing | 2 | 119 references cited in author-date format, in the reference style of the journal. Sources are drawn from primary peer-reviewed literature where available, spanning foundational posture neurophysiology, primary spinal and brainstem electrophysiology and pharmacology, anaesthesia case and volunteer literature on opioid-induced rigidity, public health surveillance, prospective clinical cohorts with toxicological confirmation, and observational and ethnographic work from supervised consumption settings. Conference abstracts and meeting proceedings were excluded (Supplementary Material 1). |
| 5 | Scientific reasoning (e.g. inclusion of appropriate evidence) | 1 | Claims are supported by sources from the evidence tier specified in Supplementary Material 2, and evidence running counter to the primary hypothesis is reported at full weight and identified as such. The item is scored 1 rather than 2 for a reason internal to the subject matter: the central mechanistic proposition concerns axial motor output during illicit fentanyl exposure, which has not been measured. The argument therefore rests on convergent inference from adjacent literatures, and its status is stated where it is advanced (§3.3). This is a limitation of the available evidence rather than of the synthesis, and it constrains what the reasoning can establish. |
| 6 | Appropriate presentation of data (e.g. graphical and tabular display) | 2 | Three tables are presented. One sets out what the evidence establishes and where it conflicts, by domain. A second sets out the conditions to be excluded and the candidate explanations to be assessed. A third gives the predicted findings under each candidate account across eight measurements. Three figures support the framework. Three supplementary files document methodology: search strategy (Supplementary Material 1), evidence hierarchy (Supplementary Material 2), and this checklist. |

**Summary**

**Self-assessed total: 11 of 12.**

Item 5 is scored 1 because the central mechanistic claim concerns a measurement that has not been made in the population of interest; the remaining items are scored 2.

**Reference**

Baethge C, Goldbeck-Wood S, Mertens S. SANRA—a scale for the quality assessment of narrative review articles. *Res Integr Peer Rev*. 2019;4:5. (Cited in the manuscript as Baethge et al. 2019.)
